# COVID-19-Associated Thrombotic Thrombocytopenic Purpura: A Case Report and Systematic Review

**DOI:** 10.3390/hematolrep14030035

**Published:** 2022-08-02

**Authors:** Haseeb Chaudhary, Usama Nasir, Khezar Syed, Maria Labra, Christopher Reggio, Ansar Aziz, Parin Shah, Roopika Reddy, Navdeep Sangha

**Affiliations:** 1 Department of Internal Medicine, Reading Hospital, Reading, PA 19611, USA; usama.nasir@towerhealth.org (U.N.); khezar.syed@towerhealth.org (K.S.); maria.labra@towerhealth.org (M.L.); christopher.reggio@towerhealth.org (C.R.); 2 Department of Hospital Medicine, Reading Hospital, Reading, PA 19611, USA; ansar.aziz@towerhealth.org; 3 Department of Pulmonary and Critical Care, Reading Hospital, Reading, PA 19611, USA; parin.shah@towerhealth.org (P.S.); roopika.reddy@towerhealth.org (R.R.); 4 Department of Hematology and Oncology, Reading Hospital, Reading, PA 19611, USA; navdeep.sangha@towerhealth.org

**Keywords:** SARS-CoV-2, COVID-19, TTP, refractory, thrombotic microangiopathies

## Abstract

Introduction: The proliferation of literature regarding the COVID-19 pandemic has served to highlight a wide spectrum of disease manifestations and complications, such as thrombotic microangiopathies. Our review with a brief case presentation highlights the increasing recognition of TTP in COVID-19 and describes its salient characteristics. Methods: We screened the available literature in PubMed, EMBASE, and Cochrane databases from inception until April 2022 of articles mentioning COVID-19-associated TTP in English language. Results: From 404 records, we included 8 articles mentioning data of 11 patients in our review. TTP was predominantly reported in females (72%) with a mean age of 48.2 years (SD 15.1). Dyspnea was the most common symptom in one third of patients (36.6%). Neurological symptoms were reported in 27.3% of cases. The time to diagnosis of TTP was 10 days (SD 5.8) from onset of COVID-19. All 11 cases underwent plasma exchange (PLEX), with a mean of 12 sessions per patient, whereas 6 cases received Rituximab (54.5%), and 3 received Caplacizumab (27.3%). One patient died from the illness. Conclusion: This review of available literature highlights the atypical and refractory nature of COVID-19-associated TTP. It required longer sessions of PLEX, with half of the patients receiving at least one immunosuppressant.

## 1. What Is the New Aspect of Your Work?

This is the first systematic review combining the available data on the rare association of COVID-19-triggered thrombotic thrombocytopenic purpura (TTP).

## 2. What Is the Central Finding of Your Work?

COVID-19-associated TTP had an atypical mode of presentation, with no patients reporting fever and only 27.3% having neurological symptoms. The development of TTP was unrelated to the severity of COVID-19 in our review, with a mean of 10 days from COVID-19 symptoms to TTP diagnosis. In terms of management, it required a mean of 12 plasma exchange sessions per patient, with more than half requiring immunosuppressive therapies. This may signify the refractory nature of TTP.

## 3. What Is (or Could Be) the Specific Clinical Relevance of Your Work?

The pathophysiology and management of TTP in COVID-19 remains an evolving field. This review of available literature highlights the atypical and refractory nature of COVID-19-associated TTP that needs early recognition and appropriate management of this life-threatening condition.

## 4. Introduction

Thrombotic thrombocytopenic purpura (TTP) is part of a broad spectrum of thrombotic microangiopathies (TMAs) that presents with significant manifestations of hemolytic anemia. The pathophysiology of this entity is endothelial dysfunction characterized by thrombocytopenia, hemolytic anemia, schistocytes, and tissue injury, which are caused by the deficiency of a disintegrin and metalloproteinase with a thrombospondin type 1 motif, member 13 (ADAMTS13), allowing the unregulated proliferation of microthrombi composed of von Willebrand factor and platelets. Only a small percentage of patients present with the characteristic pentad of microangiopathic hemolytic anemia (MAHA), thrombocytopenia, neurologic manifestations, fever, and renal failure [1]. The central nervous system and the kidneys are the two organ systems most afflicted by TTP [1].

The Oklahoma TTP-HUS registry approximates the incidence of TTP as 3 in 1 million adults per year, with a median age of diagnosis of 41 years [2]. TTP is a medical emergency with a 90% death rate if not diagnosed and treated promptly. It can be challenging to diagnose immune-mediated TTP since its clinical manifestations are similar to those of other TMAs, including moderate to severe thrombocytopenia, MAHA, and indications of ischemia and end-organ destruction. Although diagnosis is made clinically, the activity of ADAMTS13, which is 10% in acute TTP, is measured to support the diagnosis [3]. However, ADAMTS13 testing is not readily available in many hospitals, and results can take several days, which may be impractical for a timely diagnosis.

As the COVID-19 pandemic rapidly spread around the globe, many scientific facts and data about the pathogenicity of this virus were discovered. Deep venous thrombosis, pulmonary thromboembolism, and TMAs are some of the conditions associated with COVID-19 coagulopathy [4]. The association between TTP and COVID-19 has been rarely described to date. This systematic review focused on data from adult patients who were diagnosed with COVID-19-associated TTP. This review aims to provide a comprehensive summary of the available evidence regarding COVID-19-associated TTP with a brief mention of a case at our healthcare center.

## 5. Case Presentation

A 33-year-old, previously vaccinated female was diagnosed with COVID-19 infection via nasopharyngeal PCR after experiencing flu-like symptoms. She did not require any treatment at that time. On day 10 from symptom onset, she presented to the hospital with increased bruising and heavy menstrual flow along with ecchymosis and petechiae on her arms and chest. She was afebrile, with a regular pulse of 89 beats/min, respiratory rate of 15 breaths/min, blood pressure of 124/79 mmHg, and saturation of 100% on ambient air. Laboratory evaluation revealed hemoglobin of 9.1 g/dL, platelets of 9.0 × 10^3^/microL, haptoglobin 30 mg/dl, LDH of 695 IU/L, bilirubin of 3.0 mg/dL, creatinine of 0.52 mg/dL, and marked schistocytes on peripheral smear with a negative Coombs test. She had negative ANA, lupus anticoagulant, CMV serology, parasite smear, HIV, Hepatitis B, and pregnancy test.

With a high clinical suspicion of immune TTP, and a PLASMIC score of 7, treatment was initiated with Prednisone 1 mg/kg/day. She also received five sessions of plasmapheresis (PLEX), and one dose of Rituximab (RTX). On day 5 of admission, her platelet count improved to 181 × 10^3^ IU/L. ADAMTS13 activity level, that had been drawn prior to starting PLEX, was 0.03% (normal 60%) and with an ADAMTS13 inhibitor value of 2.3 BU (normal 0.5 BU).

She was discharged from the hospital on a Prednisone taper. Follow-up in our clinic the following week showed a decline in platelet count to 85 × 10^3^ IU/L. Repeat ADAMTS13 level was still undetectable, and ADAMTS13 inhibitor was 1.4 BU. Patient was readmitted and reinitiated on PLEX and RTX with the addition of a pulse dose of methylprednisolone of 1 g daily for 3 days, followed by 1 mg/kg of Prednisone daily, as well as Caplacizumab (CZB). After the fifth day, platelets improved to over 150 × 103 IU/L and she was discharged with a plan to complete CZB daily for thirty days along with Rituximab for a total of four doses, with successful recovery. 

## 6. Methods

### 6.1. Search Strategy

We performed a comprehensive database search on MEDLINE (via PubMed), EMBASE, and Cochrane databases for published articles describing the association of COVID-19 and TTP from inception until February 2022. The search strategy was performed using a concise vocabulary, as shown in Supplementary File S1. The search strategy was designed and performed by an experienced librarian with input from the principal investigator. We limited our search to articles published in English language only.

### 6.2. Study Selection and Characteristics

**Figure 1:**
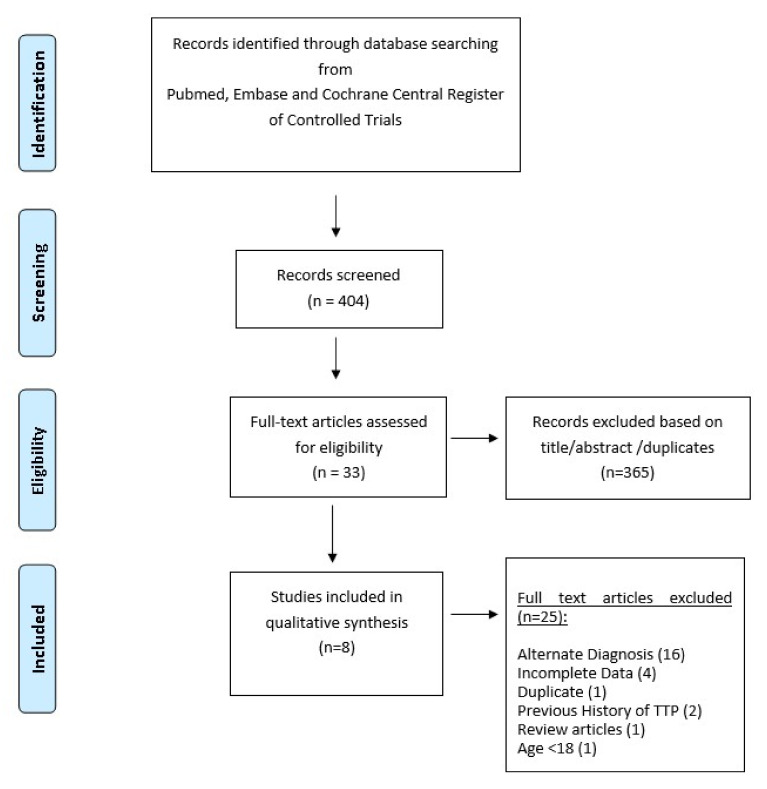
Preferred Reporting Items for Systematic Reviews and Meta-Analyses (PRISMA) flow diagram of included articles.

All articles in English were screened by two authors individually, H.C. and U.N. The extracted articles were screened by their title and abstract. Articles not meeting the inclusion criteria or without confirmation of TTP with ADAMTS13 levels were not included. We restricted our inclusion to adults aged 18 and older. We excluded the articles reporting a relapse of TTP in previously diagnosed immune TTP. We also excluded articles reporting TTP associated with COVID-19 vaccination. Full-text articles were reviewed after the initial screening. Commentaries, review articles, and abstracts without full texts were excluded as well. We included 8 articles including the data of 11 patients with COVID-19-associated TTP [3, 5,6,7,8,9,10,11], as depicted in the PRISMA flow diagram in Figure 1.

### 6.3. Quality Assessment of Articles

All the included articles were case reports and series. The case reports were assessed for quality by using the Joanna Briggs Index (JBI) Critical Appraisal Checklist for Case Reports, available at https://jbi.global/sites/default/files/2019-05/JBI_Critical_Appraisal-Checklist_for_Case_Reports2017_0.pdf (accessed on 3 February 2022) (Supplementary File S2).

## 7. Results

### 7.1. Patient Characteristics

As depicted in Table 1, the cases of COVID-19-induced TTP had a female predominance (*n* = 8, 72.7%) with a mean age of 48.4 years (SD 15.1 years). Two cases had a previous diagnosis of locally advanced breast cancer in remission and one patient was pregnant. Dyspnea was the most common symptom at presentation, present in more than one third of the patients (*n* = 4, 36.6%), with neurological symptoms being the second most common (*n* = 3, 27.3%, of whom two presented with seizures (18.2%). One patient presented with back pain, while one patient presented with rectal bleeding. None of the patients had febrile illness with onset of TTP. The mean duration from the onset of COVID-19 to diagnosis of TTP was 10 days (SD 5.8).

### 7.2. Laboratory Parameters

The mean hemoglobin (Hb) at the time of TTP diagnosis was 7.6 g/dL (SD 2.1), mean platelet level was 25.8 × 10^3^/microL (SD 26.3 × 10^3^), and median LDH level was 1422 U/L (range: 545–10,977 U/L). All the included cases (11) had a reported ADAMTS13 activity of less than 10%. Eight (72%) cases had elevated ADAMTS13 inhibitor or antibody levels, with the exception of three cases [5,10,11]. The pertinent laboratory investigations with other inflammatory markers and radiological findings are as summarized in Table 2.

### 7.3. Treatment

All 11 cases underwent PLEX, with an average number of 12 sessions (SD 4.36) per patient (n = 10). All patients received steroids, six patients (54.5%) received Dexamethasone with an average dose of 16 mg/day (equivalent Methylprednisone dose = 85.3 mg/day), and five patients (41.7%) received Prednisone/Methylprednisone with an average dose of 1 mg/kg/day. Two cases did not mention the steroid dosage used. Out of the 11 cases, 6 received RTX (54.5%) [3,6,8,9,11], while 3 received CZB (27.3%), including 2 cases that received it in addition to RTX [8,9] (Table 2). Almost half of the patients did not receive additional immunosuppressants with PLEX despite more than five sessions of PLEX.

### 7.4. Outcome

Majority of cases (90.9%) had a favorable outcome with no mention of early relapse. There was one reported death [11]. The median length of hospital stay was 18.5 days (7–51). The duration of ICU stay was only reported in two cases, with one patient requiring three weeks of intensive care.

### 7.5. Discussion 

Our review highlights the increasing recognition of TTP in COVID-19 infections and summarizes important features of COVID-19-associated TTP. TTP was predominantly reported in females (72%), and neurological symptoms were present in 27.3% of cases, with absence of fever at the time of TTP diagnosis amongst all. The mean number of days for diagnosis of TTP was 10 days (SD 5.8) from the onset of COVID-19 symptoms. The type and dose of steroid therapy was inconsistent in majority of cases, though more than half received Dexamethasone 16 mg/day. All 11 cases underwent PLEX, with a mean of 12 sessions (SD 4.36) per patient, whereas 6 cases received RTX (54.5%), and 3 cases received CZB (27.3%) in addition to PLEX and corticosteroid therapy.

These findings may suggest a refractory nature of COVID-19-associated TTP. The current practice for management of acquired TTP is to perform at least five sessions of PLEX or until platelet and hemolysis parameters normalize for two consecutive days [12,13]. The cases requiring more than 4–7 sessions are labeled as refractory. The relapse rate in acquired TTP varies from 10% to 40%, with response rates of nearly 100% in those who receive RTX shown in a few observational studies [14]. These findings have not been replicated in our review. Although majority of the cases required more than five days of PLEX, nearly half of them received RTX. The lack of robust data favoring this approach and concomitant COVID-19 infection may have guided decision-making in these cases, as effects of RTX on COVID-19 infection can be detrimental. Interestingly, clinicians opted to use RTX in two of the reported cases despite severe COVID-19 infection, and this had a favorable outcome in one [9,11].

CZB, a novel monoclonal therapy that inhibits the binding of vWF to glycoprotein 1b present on platelets, has been increasingly used as part of the treatment strategy for severe TTP. CZB has been shown to decrease rates of relapse and time to platelet recovery in a randomized double-blind study [15]. Our review showed that three cases (27.3%) required CZB, with triple therapy comprising PLEX, RTX, and CZB in only two of these [8,9,10]. In 1 case by Nicolotti et al., CZB was added owing to the inadequate response after 11 PLEX sessions and 2 doses of RTX. One case by Law et al. also had minimal response to frontline RTX, leading to the addition of CZB with PLEX on day 17 [8]. None of the cases from this review mentioned an early relapse on follow-up. However, our patient had an early relapse with five sessions of PLEX and two weeks of RTX therapy, requiring the addition of CZB. The triple therapy is supported by a recent prospective study supporting the role of RTX and CZB as complementary in the management of acquired TTP [16]. Another consideration in the management of refractory TTP cases in COVID-19 infection may be widespread activation of complement-mediated endothelial injury causing micro-thrombosis. One series of five cases showed evidence of complement deposition in vascular tissue without viral cytopathic effects on endothelium [17]. These cases can be difficult to differentiate from acquired TTP and appear to be more consistent with a COVID-19-induced complement-mediated TMA. In these cases, the role of complement inhibition may be effective. However, this was not reported in any of our reviewed cases.

Although a causal relationship remains to be elucidated, viral infections are a known trigger for secondary or immune TTP, with proposed mechanisms including both direct endothelial injury and creation of ADAMTS13 autoantibodies [18,19]. The hypercoagulable state conferred by COVID-19 has also been postulated to be direct cytopathic injury to endothelial cells mediated by entry via ACE-2 receptors as well [20]. Small-scale studies have also shown decreased ADAMTS13 levels secondary to COVID-19 inflammation [21]. We hypothesize that these multiple mechanisms may explain the refractory nature of COVID-19-associated TTP. Two recent studies by Mancini et al. [21] and Pascreau et al. [22] including 120 patients showed the correlation of decreased ADAMTS13 activity with the severity of COVID-19 illness. Interestingly, most reviewed cases (81.8%) with confirmed TTP had undetectable ADAMTS13 activity (i.e., 10%) without clinically severe COVID-19 infection.

The combination of elevated vWF, Factor VIII, fibrinogen, and D-dimer levels in COVID-19 infections has also been noted in studies [23]. This in turn has led to a spectrum of thrombotic disorders including COVID-19-induced micro-thrombotic disease, which differs from TTP or other primary TMAs in the absence of MAHA and thrombocytopenia. When present, severe thrombocytopenia and hemolytic anemia could be representative of disseminated intravascular coagulation (DIC), HUS, or drug-related TMA, which are also not uncommonly noted in COVID-19 infections [24]. These often clinically overlapping conditions highlight the need for prompt recognition of TTP as mortality in untreated patients can be over 90% and 10% if appropriate timely therapy is initiated [25].

This is the first systematic review combining all the data regarding disease characteristics and outcomes of confirmed TTP cases in COVID-19 patients, to the best of our knowledge. However, we do note some limitations. First, none of the articles clearly mentioned if the patients were unvaccinated against COVID-19. This is important because the development of TTP secondary to COVID-19 vaccination has been previously reported. Whether the previous immunity against COVID-19 prevents the development of TTP after disease exposure is unknown. Second, the characteristics of COVID-19 severity were also not clearly defined in some of the articles. It may be worth exploring if severe COVID-19 infection is a risk factor for development of TTP as severe endothelial inflammation leading to vWF release may have a role to play. Finally, an inherent limitation is the rarity of this association, with only a small number of cases reported to date, which warrants further large-scale studies.

## 8. Conclusions

In summary, the pathophysiology and management of TTP in COVID-19 remains an evolving field. This review of available literature highlights the atypical and refractory nature of COVID-19-associated TTP. It required longer sessions of PLEX, with half of the patients receiving at least one immunosuppressant.

## Figures and Tables

**Table 1:** Patient symptomatology, time to diagnosis, and treatment of TTP.

Study/Year	Age (Years)/Gender	Comorbidities	Symptoms	Time from COVID-19 Illness to TTP Diagnosis	Steroids (Prednisone Equivalent Dose in mg/Day)	PLEX	RTX	CZB
**Albiol et al. 2021 [5]**	57/F	Hypertension Breast Cancer in remission	Dry cough Anosmia Dysgeusia	Day 6	Methylprednisone 1 mg/kg	8 sessions	Yes	No
**Beaulieu et al. 2021 [6]**	70/M	Peripheral arterial disease, dyslipidemia	Confusion, seizure, dark urine	Day 19	Methylprednisone 1 mg/kg	7 sessions	No	No
**Dhingra et al. 2021 [7]**	35/F	None	Diarrhea, right hemiparesis, seizure	Day 15	Methylprednisolone 1 g injection	16 sessions	Yes	No
**Hindilerden et al. 2020 [8]**	74/F	HTN	Dry cough, Fatigue	Day 5	Methylprednisolone 1 mg/kg/day	11 sessions	No	No
**Law 2021 et al. [9]**	47/F	None	Fatigue, scleral icterus, dark urine	Day 17	Dexamethasone (dose not mentioned)	3 sessions	Yes	Yes
**Nicolotti et al. 2021 [10]**	44/F	Obesity, Hx of DVTs	Weakness, dizziness, abdominal discomfort, respiratory distress	Day 3	Methylprednisolone (1 mg/kg, 5 days)	14 sessions	Yes	Yes
**Shankar et al. 2021 [11]**	30/M	None	Low back pain, left flank pain, hematuria	Day 7	Prednisone 1 mg/kg/day	6 sessions	No	Yes
**Tehrani 2021 [12]**								
**(i)**	25/F	Pregnant	Severe respiratory symptoms	Not mentioned	Dexamethasone 8 mg BID daily, 14 days	10 days of sessions	No	No
**(ii)**	56/F	Locally advanced breast cancer/In remission	Severe respiratory symptoms	Not mentioned	Dexamethasone 8 mg BID daily, 14 days	14 days of sessions exchange	Yes	No
**(iii)**	57/F	None	Severe respiratory symptoms	Not mentioned	Dexamethasone 8 mg BID daily, 14 days	14 sessions	No	No
**(iv)**	38/M	None	Rectal bleeding	Not mentioned	Dexamethasone 8 mg BID, 21 days	21 sessions	Yes	No

(CZB = Caplacizumab, FFP = fresh frozen plasma, PLEX = plasmapheresis, RTX = Rituximab).

**Table 2:** Laboratory findings in patients with COVID-19-associated TTP.

Study/Year	Hb in g/dL	Plt Count × 10^3^/m L	I NR	LDH (U/L)	ADAMTS13 Activity Ag (%)	ADAMTS13 Inhibitor Level (normal 12 U/mL; 0.5 BU)	ADAMTS13 Antibody Titer (Normal 15 U/mL)	CT/CXR of Lung Findings
**Albiol et al. 2021 [5]**	6.9	13	N/A	1594	2.0%	5.2 BU	-	CT thorax normal
**Beaulieu et al. 2021 [6]**	6.0	18	1.1	1422	10%	-	0.5	CXR normal
**Dhingra et al. 2021 [7]**	8.3	20	N/A	10,977	undetectable	3.0 BU	-	n/a
**Hindilerden et al. 2020 [8]**	6.6	48	Normal	1108	0.2%	90 U/mL	-	Patchy peripheral bibasilar ground glass opacities in both lungs. MRI brain normal
**Law et al. 2021 [9]**	7.0	14	1.1	788	5.0%	63 U/mL	-	n/a
**Nicolotti et al. 2021 [10]**	6.0	7	Normal	2961	5.0%	57 U/mL	-	Interstitial pneumonia involving 25% of lung parenchyma
**Shankar et al. 2021 [11]**	13.7	9	Normal	1068	3.0%	0.60 BU	-	n/a
**Tehrani et al. [12]**								
**(i)**	7.0	10.5	1.4	3465	8.0%	-	85	Patchy infiltration
**(ii)**	6.0	41	1.2	1520	0.01%	-	36.2	Patchy bilateral infiltration
**(iii)**	7.9	98	1.5	1150	0.86%	-	25.3	Not mentioned
**(iv)**	8.0	5.0	1.3.	545	0.06%	-	14	Patchy infiltration in the right upper lobe

ADAMTS13 = A disintegrin and metalloproteinase with a thrombospondin type 1 motif, member 13; CT = computed tomography; BU = Bethesda unit; CXR = chest X-ray; CRP = C-reactive protein; Hb = hemoglobin; INR = international normalized ratio; LDH = lactate dehydrogenase, Plt = platelets.

## Data Availability

The authors declare that data supporting the findings of this study are available online.

## Supplementary Materials

The following supporting information can be downloaded at: https://www.mdpi.com/article/10.3390/hematolrep14030035/s1, File S1: Search Strategy; File S2: grading of studies.
