# Supplementary material for: COVID-19-Associated Thrombotic Thrombocytopenic Purpura: A Case Report and Systematic Review
Source: Hematol Rep. 2022 Aug 2;14(3):253–60. doi: 10.3390/hematolrep14030035 (PMC9397065; doi:10.3390/hematolrep14030035)
Supplement: Supplementary file 1 [file hematolrep-14-00035-s001.zip › Supplementary File 1. Search Strategy.pdf]

EMBASE 192

('coronavirus disease 2019'/exp OR '2019 novel coronavirus disease' OR '2019 novel coronavirus epidemic' OR '2019 novel coronavirus infection' OR '2019-ncov disease' OR '2019-ncov infection' OR 'covid' OR 'covid 19' OR 'covid 19 induced pneumonia' OR 'covid 2019' OR 'covid-10' OR 'covid-19' OR 'covid-19 induced pneumonia' OR 'covid-19 pneumonia' OR 'covid19' OR 'sars coronavirus 2 infection' OR 'sars coronavirus 2 pneumonia' OR 'sars-cov-2 disease' OR 'sars-cov-2 infection' OR 'sars-cov-2 pneumonia' OR 'sars-cov2 disease' OR 'sars-cov2 infection' OR 'sarscov2 disease' OR 'sarscov2 infection' OR 'wuhan coronavirus disease' OR 'wuhan coronavirus infection' OR 'coronavirus disease 2' OR 'coronavirus disease 2010' OR 'coronavirus disease 2019' OR 'coronavirus disease 2019 pneumonia' OR 'coronavirus disease-19' OR 'coronavirus infection 2019' OR 'ncov 2019 disease' OR 'ncov 2019 infection' OR 'new coronavirus pneumonia' OR 'novel coronavirus 2019 disease' OR 'novel coronavirus 2019 infection' OR 'novel coronavirus disease 2019' OR 'novel coronavirus infected pneumonia' OR 'novel coronavirus infection 2019' OR 'novel coronavirus pneumonia' OR 'paucisymptomatic coronavirus disease 2019' OR 'severe acute respiratory syndrome 2' OR 'severe acute respiratory syndrome 2 pneumonia' OR 'severe acute respiratory syndrome cov-2 infection' OR 'severe acute respiratory syndrome coronavirus 2 infection' OR 'severe acute respiratory syndrome coronavirus 2019 infection') AND ('thrombotic thrombocytopenic purpura'/exp OR 'ttp (disease)' OR 'essential thrombocytopaenia' OR 'essential thrombocytopenia' OR 'purpura, thrombotic thrombocytopenic' OR 'thrombocytopaenia, thrombotic' OR 'thrombocytopenia, thrombotic' OR 'thrombocytopenic purpura, thrombotic' OR 'thrombocytopenic thrombotic purpura' OR 'thrombotic thrombocytopaenia' OR 'thrombotic thrombocytopenia' OR 'thrombotic thrombocytopenic purpura') AND 'diagnosis'/lnk

.

PubMed 155 results

("Autoimmune thrombotic thrombocytopenic purpura"[All Fields] OR "TTP"[All Fields] OR "Thrombotic Thrombocytopenic Purpura"[All Fields] OR "iTTP"[All Fields] OR "thrombotic microangiopathy"[All Fields] OR "TMA"[All Fields] OR "purpura, thrombotic thrombocytopenic/diagnosis"[MeSH Terms] OR "moschcowitz disease"[Title/Abstract] OR "immune thrombotic thrombocytopenic purpura"[Title/Abstract]) AND (("COVID-19"[All Fields] OR "COVID-19"[MeSH Terms] OR "COVID-19 Vaccines"[All Fields] OR "COVID-19 Vaccines"[MeSH Terms] OR "COVID-19 serotherapy"[All Fields] OR "COVID-19 serotherapy"[Supplementary Concept] OR "covid 19 nucleic acid testing"[All Fields] OR "covid 19 nucleic acid testing"[MeSH Terms] OR "covid 19 serological testing"[All Fields] OR "covid 19 serological testing"[MeSH Terms] OR "covid 19 testing"[All Fields] OR "covid 19 testing"[MeSH Terms] OR "sars cov 2"[All Fields] OR "sars cov 2"[MeSH Terms] OR "Severe Acute Respiratory Syndrome Coronavirus 2"[All Fields] OR "NCOV"[All Fields]

OR "2019 NCOV"[All Fields] OR (("coronavirus"[MeSH Terms] OR "coronavirus"[All Fields] OR "COV"[All Fields]) AND 2019/11/01:3000/12/31[Date - Publication])) AND ("diagnos*"[All Fields] OR "detect*"[All Fields] OR "diagnosis"[MeSH Terms] OR "diagnostic equipment"[MeSH Terms] OR "diagnostic errors"[MeSH Terms] OR "diagnostic imaging"[MeSH Terms] OR "diagnostic services"[MeSH Terms] OR "diagnosis, differential"[MeSH Terms] OR "diagnosis"[MeSH Subheading]))

Cochrane Database – 0 results as no clinical trials on this topic.
