# Supplementary material for: COVID-19-Associated Thrombotic Thrombocytopenic Purpura: A Case Report and Systematic Review
Source: Hematol Rep. 2022 Aug 2;14(3):253–60. doi: 10.3390/hematolrep14030035 (PMC9397065; doi:10.3390/hematolrep14030035)
Supplement: Supplementary file 1 [file hematolrep-14-00035-s001.zip › Supplementary File 2. Grading of studies.pdf]

**Table S2. Major**				
**P. The Joanna Briggs Institute (JBI) Critical Appraisal Checklist for Case Reports (last amended in 2017)**<br><br>**Website:** https://joannabriggs.org/critical_appraisal_tools<br><br>https://wiki.joannabriggs.org/display/MANUAL/Appendix+7.4+Critical+appraisal+checklist+for+case+reports				
Major Components	Response options			
1. Were patient's demographic characteristics clearly described?	Yes	No	Unclear	Not applicable
2. Was the patient's history clearly described and presented as a timeline?	Yes	No	Unclear	Not applicable
3. Was the current clinical condition of the patient on presentation clearly described?	Yes	No	Unclear	Not applicable
4. Were diagnostic tests or assessment methods and the results clearly described?	Yes	No	Unclear	Not applicable
5. Was the intervention(s) or treatment procedure(s) clearly described?	Yes	No	Unclear	Not applicable
6. Was the post-intervention clinical condition clearly described?	Yes	No	Unclear	Not applicable
7. Were adverse events (harms) or unanticipated events identified and described?	Yes	No	Unclear	Not applicable
8. Does the case report provide takeaway lessons?	Yes	No	Unclear	Not applicable
Overall appraisal: Include ☐ Exclude ☐ Seek further info ☐				
10. Were uninterpretable/ intermediate test results reported? (uninterpretable results reported)	Yes	No	Unclear	
11. Were withdrawals from the study explained? (withdrawals explained)	Yes	No	Unclear	
